# Lupin-Derived Bioactive Peptides: Intestinal Transport, Bioavailability and Health Benefits

**DOI:** 10.3390/nu13093266

**Published:** 2021-09-18

**Authors:** Innocent U. Okagu, Joseph C. Ndefo, Emmanuel C. Aham, Joy I. Obeme-Nmom, Precious E. Agboinghale, Rita N. Aguchem, Regina N. Nechi, Carmen Lammi

**Affiliations:** 1 Department of Biochemistry, University of Nigeria, Nsukka 410001, Nigeria; innocent.okagu@unn.edu.ng (I.U.O.); emmanuel.aham@unn.edu.ng (E.C.A.); aguchemrita@gmail.com (R.N.A.); 2 Department of Science Laboratory Technology, University of Nigeria, Nsukka 410001, Nigeria; 3 Department of Biochemistry, College of Pure and Applied Sciences, Landmark University, PMB 1001, Omu-Aran 251101, Nigeria; obemejoy@gmail.com; 4 Department of Biochemistry, Afe Babalola University, Ado-Ekiti 360001, Nigeria; preciousfredricks1@gmail.com; 5 Faculty of Pharmaceutical Sciences, University of Nigeria, Nsukka 410001, Nigeria; amaka.nechi@gmail.com; 6 Department of Pharmaceutical Sciences, University of Milan, Via Mangiagalli 25, 20133 Milano, Italy

**Keywords:** lupin, *Lupinus species*, lupin protein hydrolysates, lupin-derived peptides, functional foods, bioactive peptides, nutraceuticals

## Abstract

There is a renewed interest on the reliance of food-based bioactive compounds as sources of nutritive factors and health-beneficial chemical compounds. Among these food components, several proteins from foods have been shown to promote health and wellness as seen in proteins such as α/γ-conglutins from the seeds of *Lupinus* species (Lupin)*,* a genus of leguminous plant that are widely used in traditional medicine for treating chronic diseases. Lupin-derived peptides (LDPs) are increasingly being explored and they have been shown to possess multifunctional health improving properties. This paper discusses the intestinal transport, bioavailability and biological activities of LDPs, focusing on molecular mechanisms of action as reported in in vitro, cell culture, animal and human studies. The potentials of several LDPs to demonstrate multitarget mechanism of regulation of glucose and lipid metabolism, chemo- and osteoprotective properties, and antioxidant and anti-inflammatory activities position LDPs as good candidates for nutraceutical development for the prevention and management of medical conditions whose etiology are multifactorial.

## 1. Introduction

For many centuries, human and animals have depended on plants and their products as source of nutrients and other chemicals that promote good health and wellness. These plant-derived chemicals include alkaloids, polyphenols, vitamins, minerals, proteins, peptides, and lipids among others [1]. In addition, epidemiological and preclinical studies show that eating a plant-based diet lowers the risk of chronic disease development [2,3]. *Lupinus* species (Lupin) is a genus of a leguminous plant that is made up of 200 species in the Fabaceae family. Among the *Lupinus* spp*., L. albus* (white lupin)*, L. angustifolius* (narrow-leaf lupin), and *L. luteus* (annual-yellow lupin) are the most consumed [4,5]. Several health-promoting properties have been reported of *Lupinus* species, mainly *L. albus* and *L. angustifolius,* such as antioxidant, anti-inflammatory, hypolipidemic, hypoglycemic, and hypotensive properties among others in several preclinical and clinical human and animal studies [6,7,8,9,10,11,12,13,14,15]. These biological activities are attributed to their human-health beneficial chemical components, such as polyphenols, carotenoids and other phytochemicals [16,17]. These multifunctional properties make lupin special candidates for industrial use in the development of functional food products like in bakery and supplement development [17,18,19]. Consumption of lupin kernel fibre-enriched snacks was shown to improved bowel function and reduce the risk to colon cancer [20]. In addition, alcoholic extracts of the shoots and roots, and the seeds have been shown to possess antiproliferative activities in both drug-sensitive and drug-resistant breast cancer cell lines [21]. Similarly, ethanolic extract of the seeds alone arrested colon cancer cells at the G2/M phase of cell cycle and induced apoptosis by upregulating the expression of pro-apoptotic genes in cultured cells exposed to the extract. Interestingly, the anti-colon cancer activity by lupin seed extract was better than fluorouracil, a known anticancer agent [22]. 

Due to the high protein content of lupin seeds (31–52%) [23,24,25,26], proteins derived from lupin seeds are gaining attention as source of bioactive peptides. Several lines of evidence highlight the biological activities of lupin protein hydrolysates (LPHs) and peptides (LDPs) including hypocholesterolemic, hypoglycemic, antimicrobial, anti-inflammatory and immunomodulatory effects [27,28,29,30,31,32,33,34,35]. The health-promoting effects of lupin proteins and hydrolysates consumptions have been tested in human clinical trials. Lupin protein-enriched diet such as bread, biscuits, and pasta interestingly suppressed the lipid profile and blood pressure in both normal and hypertensive human subjects [5,8,33,36,37]. Similarly, lupin-based diets improved glycemic control in diabetic and normal human subjects [38,39,40,41]. In addition, a recent study supports the pleotropic actions of a lupin bioactive peptides-based functional food on key steps of atherosclerosis including inflammation, oxidative stress, and cholesterol metabolism [42]. In this context, lupin hydrolysates stand out also for their powerful content of multifunctional peptides, i.e., peptides that have the capacity to impart more than one physiological outcome by affecting different targets compared to monofunctional peptides [43]. 

In the field of legumes, many pieces of evidence highlight the health-promoting effects of peptides which are generated by the hydrolysis of proteins derived by other legumes, i.e., soyabean (*Glycine max*), peas (*Pisum sativum*), chickpea (*Cicer arietinum)*, and beans (*Phaseolus vulgaris*) [44]. In general, hydrolysates from the same source may contain many peptides, which can present different biological activities depending on the enzymes used, the enzyme:substrate ratio, and the hydrolysis time [44]. More in details, many legume derived peptides are described possessing single or multifunctional biological activities exerting hypocholesterolemic, hypotensive, antioxidant, and antitumor effects [45]. Indeed, a peptide from soybean glycinin, i.e., LPYP, and four peptides from soybean β-conglycinin; i.e., IAVPTGVA, IAVPGEVA, YVVNPDNDEN and YVVNPDNNEN, modulate the cholesterol metabolism through the direct ability to inhibit the HMGCoAR which in turn lead to the increase of LDLR protein level and its activity in HepG2 cells [46,47]. In addition, LPYP, IAVPTGVA, and IAVPGEVA display anti-diabetic activities through the modulation of both Akt and AMPK-pathway, respectively, which induce the augmentation of GLUT1 and GLUT4 transporter on HepG2 cell membranes [48]. The peptide FVVNATSN has been identified as able to increase the LDL-receptor mRNA in HepG2 cells [49]. VAWWMY is a glycinin derived peptide, named soy statin, which acts as inhibitor of cholesterol absorption in vivo [50]. Soybean is also a rich source of lunasin (a unique 43-amino acid polypeptide sequence encoded within the soybean Gm2S-1 gene), which possess anticancer activity, antioxidant, hypocholesterolemic, and anti-inflammatory activities [51,52,53,54,55,56]. Chickpea (*Cicer arietinum*), field pea (*Pisum sativum*), mung bean (*Vigna radiata*), and kidney bean (*Phaseolus vulgaris*) among others, have yielded ACE-I inhibitory peptides. In this context, some pea protein derived dipeptides, i.e., GF, IR, and LF, exert ACE-inhibitory activity [57]. Interestingly, in a structure-activity relationships study, Wu et al. predicted the sequence of some peptides with theoretical ACE-inhibitory activity, demonstrating that two of these peptides, naturally present in pea protein primary sequences, are potent ACE-inhibitors [58].

Although some proteins are bioactive and can elicit some biological effects in their intact native nature, a majority of the proteins that are biologically inactive are encrypted with health-promoting peptides that only become active when separated from the whole protein matrix [59]. These proteins are hydrolyzed either by microbial fermentation or by using proteases; in many cases, a combination of two or more enzymes with different cleavage sites such as Neutrase, pepsin, pancreatin, flavourzym, Izyme AL and Alcalase 2.4 L to produce short chain, low molecular weight peptides. A good number of procedures and techniques such as gel filtration-coupled high performance liquid chromatography-tandem mass spectrometry (HPLC-MS), liquid chromatography-mass spectrometry (LC-MS), ultra-high performance liquid chromatography-tandem mass spectrometry (UPLC-ESI-MS/MS), ultrasound, matrix-assisted laser desorption/ ionization time-of-flight mass spectrometry (MALDI-TOF-MS), ion exchange chromatography and reversed-phase high performance liquid chromatography-tandem ultrafast liquid chromatography (RP-HPLC UFLC) [60,61] are currently being explored for the isolation, identification and quantification of peptides from protein hydrolysates. For more information on the preparation and quantification of peptides from proteins, see previous reviews [62,63,64,65,66,67].

Considering the unique nature of lupin-based peptides, this review takes into consideration all studies reporting the biological activities of lupin-derived peptides (LDPs) with special references on their molecular mechanisms of action and structure-activity relationship. In addition, the transepithelial transport, biostability, bioavailability and safety concerns of LDPs were discussed, while strategies to improve the bioavailability and biostability were also suggested. 

## 2. Biological Activities of LDPs

The health benefits of LDPs in relation to hypertensive/anti-hypertensive, anti-inflammatory, antioxidant, cytoprotective, immunomodulatory, hypoglycemic, hypo-lipidemic, osteoprotective, and neuroprotective properties are discussed below.

### 2.1. Antioxidant and Cytoprotective Effects 

The excessive production of free radicals, including reactive oxygen (ROS) and reactive nitrogen (RNS) species beyond the body’s antioxidant capacity to attenuate them results in a condition known as oxidative stress. This condition has been identified to play key roles in the initiation and progression of many disease conditions such as inflammatory bowel, cardiovascular, cerebrovascular and neurodegenerative diseases, many types of cancers [68,69], and age-related macular degeneration (AMD) [70,71]. To protect the human body from the wraths of free radicals generated from external and internal sources, the modification of dietary lifestyle to include the consumption of foods rich in radical-scavenging and antioxidant chemicals is highly encouraged [72,73]. Among the food-based chemicals that block free radical-generated damages, plant-derived polyphenols and food protein-derived bioactive peptides stand out [74]. A quadrapeptide, FVPY isolated from LPHs has been shown to have good antioxidant properties by scavenging ROS (superoxide anion and hydroxy radicals) and RNS (2,2-azino-bis(3-ethylbenzthiazoline-6-sulfonic) acid (ABTS) and 2,2-diphenyl-1-picrylhydrazyl (DPPH)) and inhibiting lipid peroxidation by greater than 70% unlike the standard antioxidant, synthetic reduced glutathione (GSH) that gave about 45% inhibition of lipid peroxidation [75]. The hydrogen atom-donating potential of tyrosine, the terminal amino acid of the peptide might have contributed to the antioxidant activity of this peptide [76].

Exposure of cells to ROS-inducers such as hydrogen peroxide (H_2_O_2_) results in the activation of kelch-like ECH-associated protein-1 (Keap-1)/nuclear factor erythroid 2-related factor 2 (Nrf2) (Keap-1/Nrf2) signaling pathways. This is done by the dissociation of the interaction between Keap-1 and Nrf2, allowing the free Nrf2 to bind to promoter region (antioxidant response elements) of genes that code for antioxidant enzymes to upregulate their expression profiles [77]. This has made Keap-1/Nrf2 signaling pathways an interesting target for attenuating many oxidative stress-related conditions, including cancers and vascular diseases [78,79]. In a study, Australian researchers showed that LMWPs derived from LPHs (6 mg/mL) with good free radical (ABTS and DPPH)-scavenging and ferric reducing antioxidant properties *in vitro*, and protected HepG2 cells from H_2_O_2_-induced oxidative damage. The LMWPs acted by suppressing intracellular ROS generation, and activating Keap-1/Nrf2 signaling pathways, leading to the increase in activities of superoxide dismutase (SOD) and glutathione peroxidase (GPX). In addition, the LMWPs upregulated gene expression profiles of genes related to antioxidant defense such as *SOD1*, *GPX1*, glutamate cysteine ligase (*GCLM*), solute carrier family7 (*SLC7A11*) and sulfiredoxin-1 (*SRXN1*) [80]. Considering that glutamate cysteine ligase is a key enzyme in the synthesis of GSH, a powerful nonenzymatic antioxidant; promotion of GSH synthesis appears to be another mechanism of action of LDPs [81]. Recently, it has also been demonstrated that the consumption of a beverage containing *L. angustifolius* protein hydrolysates by healthy subjects is able to modify the PBMCs antioxidant status assessed by TAC and ORAC assays, respectively [42]. 

In another model of inhibition of oxidative stress, GPETAFLR, a lupin-originated octapeptide, was demonstrated to increase cell viability and improve GSH synthesis, while suppressing ROS generation in retinal pigment epithelium cells [28]. This finding suggests that consumption of lupin-rich diet will provide health benefits such as delaying the onset of AMD, a condition that has been shown to be associated with free radical attack on ocular tissues, compromising the blood-retinal barrier that results in initiation and progression of ADM [82,83,84]. Taken together, these reports on the antioxidant properties of LDPs strongly position LDPs as candidates for functional food developments for diseases with oxidative stress etiology. To circumvent the limitation of biostability and bioavailability generally observed post-ingestion of peptides, the use of biocompatible carriers to deliver peptides are recommended. For instance, preparation of α-conglutin, a lupin-originated polypeptide, as a hydrogel has been shown to enhance its radical scavenging property [85,86], supporting the opinion that the practical-scale application of the multifunctional potentials of lupin-derived peptides are currently being explored.

### 2.2. Anti-Inflammatory and Immunomodulatory Effects

Due to their continued interaction with biotic and abiotic factors, human cells are exposed to inducers of inflammation, a network of reactions of immune system cells designed to eliminate sources of harm. In some situations, humans lose the capacity to control this reaction and its long-term existence results in a chronic systemic inflammatory state [87]. Uncontrolled and prolonged inflammatory processes are implicated in the etiology of many chronic conditions, such as cancer, asthma, diabetes mellitus, chronic kidney, cardiovascular, osteo-degenerative, and non-alcoholic fatty liver diseases as well as autoimmune and neurodegenerative disorders [88,89,90]. To abrogate the burden of inflammatory diseases in man, a number of interventions are already in place, such as the use of anti-inflammatory drugs and natural compounds [91,92].

Using a cell culture model, LPHs have been shown to modulate the immunological response of human-derived peripheral blood mononuclear cells treated by suppressing proliferation of the cells and level of pro-inflammatory makers (Th1, Th9 and Th17) and increasing anti-inflammatory marker (Th2), culminating into increase in anti-/pro-inflammatory status. In addition, the hydrolysates improved the antioxidant status by increasing the activities of SOD and catalase, and total antioxidant capacity [93], making their peptides potential candidates for managing disease with both inflammation and oxidative stress etiology. This in vitro evidence has also been confirmed by clinical studies. In fact, the 4-week daily intake of a functional beverage based on LPHs was reported to be safe and effective in both reducing the production of Th1 pro-inflammatory cytokines and increasing the anti-inflammatory/pro- inflammatory microenvironment of the PHA-stimulated PBMCs [42]. In a cell culture study, LPHs were shown to increase the cellular viability and expression of anti-inflammatory gene (chemokine (C-C motif) ligand 18 (CCL18)) in phorbol-12-myristate-13-acetate (PMA) activated THP-1-derived macrophages. On the other hand, the hydrolysates downregulated the expression of pro-inflammatory cytokines (tumor necrosis factor (TNF)-α and interleukins (IL)-1β, and IL-6) and their receptor, C-C chemokine receptor type 2 (*Ccr2*) and reduced nitric oxide (NO) release [94]. Generally, the reduction in NO concentration and its activity are associated with down-regulation of messenger ribonucleic acid (mRNA) expression of inducible nitric oxide synthase (*iNOS*) gene which codes for the enzyme, iNOS. This enzyme converts L-arginine to NO and citrulline [95]. NO binds to its target gene to elicit inflammatory response [96,97], making its reduction a good target of anti-inflammatory agents. Hence, the reduction in NO release by the hydrolysates is an indication of inflammation.

In immune cells, such as macrophages and monocytes, lipopolysaccharide (LPS) upregulates the gene expression profiles and activities of iNOS and cyclo-oxygenase (COX)-2, the enzymes that is centrally involved in the synthesis of NO and some inflammatory mediators known as prostaglandins (PG), respectively via interferon-gamma (IFN-γ) signaling pathway [98,99,100]. This makes LPS-activated macrophages a good model for studying anti-inflammatory properties of drugs [101]. Gao and colleagues investigated theanti-inflammatory properties of IQDKEGIPPDQQR from extruded lupin seed proteins in LPS-induced mouse RAW 264.7 macrophages [31]. The peptide increased the cell viability whereas it reduced the levels of pro-inflammatory cytokines such as monocyte chemoattractant protein-1 (MCP-1), TNF-α, IL-1β and IL-6 via downregulation of gene expression profile of p38 mitogen-activated protein kinase (MAPK) and cytokine receptors (*Tlr4* and *Ccr2*). Additionally, the peptide suppressed NO production, probably by suppressing the gene expression of iNOS [31]. In LPS-generated inflammation of retinal pigment epithelium cells (ARPE-19 cells), a lupin-derived octapeptide, GPETAFLR downregulated gene expression profile and protein levels of VEFG, IL-1β, IL-6, IFNγ, and TNF-α [28], indicating that it has anti-inflammatory properties. Taken together, the above findings show that lupin-derived peptides modulate immune response by restoring inflammatory and redox balance. These results further strengthen the opinion that LDPs may have promising applications in the prevention and treatment of inflammatory diseases. 

### 2.3. Osteoprotective Effect

As an inducer of inflammation, LPS also increases the gene expression of nuclear factor kappa B (NF-ĸB) which promotes the gene expression and production of inflammatory cytokines (TNF-α, IL-1β, and IL-6), leading to monocyte activation and the consequent induction of osteoclastogenesis (OG-differentiation of osteoclasts), a condition that increases bone resorption, osteoporosis, and other bone diseases [102,103]. This occurs through elevated expression of cytokines such as receptor activator for nuclear factor-κB (RANK) ligand (RANKL) and macrophage colony-stimulating factor (M-CSF)-induced downregulation of expression of osteoprotegerin, a protein known to protect the bones by suppresses the RANKL’s ability to induce OG and survival of bone-resorbing osteoclasts [104,105]. In LPS-induced OG in human monocyte-derived osteoclasts, a LDP (GPETAFLR) inhibits the activity of TRAP, an enzyme crucial in OG, downregulates the expression of TRAP, OSCAR, RANK, and CATHK genes, and reduces the levels of TNF-α, IL-1β, and IL-6. The peptide also upregulates the gene expression of osteoprotegerin and elevates the levels of anti-inflammatory cytokines, IL-4 and IL-10 [27]. These findings demonstrate that based on their anti-inflammatory properties, LDPs are beneficial to bone health and, hence, strengthen the existing knowledge that anti-inflammatory agents such as IL-12 [106] and non-steroidal anti-inflammatory drugs [107] inhibit OG and other bone degenerations. 

### 2.4. Neuroprotective Effect

The brain is highly susceptible to inflammation and oxidative stress due to its high lipid content; hence, the pathogenesis of many mental health and neurodegenerative diseases can in part be traced to neuroinflammation and oxidative stress [108,109,110,111,112,113]. Considering the link between inflammation, oxidative stress and brain disorders, consumption of food rich in chemicals that protect against neuroinflammation may be beneficial to mental health and neuronal wellbeing [114]. Recently, Lemus-Conejo et al. [115] have demonstrated that GPETAFLR offers protection against inflammation in BV-2 microglial cells exposed to LPS by suppressing the gene expression of M1 phenotype of peripheral macrophage system (Ccr7 and iNOS) that are responsible for the increased release of NO and pro-inflammatory cytokines. On the other hand, this peptide upregulated M2 phenotype of peripheral macrophage system (Arg-1 and Ym-1 genes) that is responsible for the production of anti-inflammatory cytokines (IL-10, IL-4, and IL-13) [114]. In obese mice induced using high-fat diet (a model known to be associated with neuroinflammation), supplementation of rodent chow with 0.5 or 1 mg/kg of GPETAFLR suppresses the total M1 phenotype microglial system leading to reduction in expression of *Ccr7* gene but not iNOS. The peptide also increases the M2 phenotype microglial system, leading to the upregulation of expression of Arg-1 and Ym-1 genes [116]. Taken together, the above findings demonstrate that GPETAFLR possesses anti-neuroinflammatory property and can further be investigated for nutraceutical development for maintaining mental health. The ability of the peptide to cross blood-brain barrier and directly exert its activity in the brain as well as the possibility of the peptide to modify microbiota population and metabolism to generate biomolecules that mediate the protection against neuroinflammation are not known and should, hence, be subject of further studies. Similarly, further investigation is required to understand if there are other metabolites of the peptide, such as intestinal proteases- and serum peptidases-generated metabolites of GPETAFLR that may be partly or entirely responsible for the observed neuroprotection. 

### 2.5. Hypocholesterolemic and Hypoglycemic Effects

Excessive production and accumulation of lipids, both in circulation and in tissues, are implicated in many conditions such as heart diseases and metabolic syndrome, both of which affect the quality of life [117]. Although statins, a class of drugs that inhibit 3-hydroxy-3-methylglutaryl CoA reductase (HMGCoAR), the key enzyme of cholesterol biosynthesis are still recommended for managing lipid levels, dietary and lifestyle modifications are highly encouraged in individuals at risk of hyperlipidemia and its comorbidities [117]. This is because of the musculoskeletal and hepatic toxicities and myopathies associated with the use of statins for controlling lipid levels [118,119,120,121,122]. LPHs elicit the complementary hypocholesterolemic effects by inhibiting the HMGCoAR activity and interaction between proprotein convertase subtilisin/kexin type 9 (PCSK9) and LDL receptor (LDLR) [35,123,124,125]. This double effect, which leads to the modulation of both LDLR and PCSK9 pathway, respectively, is due to the heterogeneous composition of the hydrolysates, in which many peptides co-exist. Interestingly, LILPKHSDAD and LTFPGSAED, from β-conglutin bind the HMGCoAR catalytic site and inhibit its enzyme activity. Indeed, biochemical and cellular studies have confirmed that these peptides are able to modulate cholesterol metabolism in a manner that leads to an increase in LDLR protein levels [125]. Briefly, both peptides inhibit in vitro HMGCoAR activity leading to an increase in LDLR protein levels due to the activation of the sterol regulatory element binding proteins (SREBP)-2 transcription factors. Moreover, through the activation of the AMPK pathway, both peptides increased the phosphorylation of HMGCoAR, decreasing its enzyme activity [125,126]. Unlike LILPKHSDAD and LTFPGSAED, the treatment of cultured human hepatic cells with YDFYPSSTKDQQS resulted in the upregulation of SREBPs-1 and LDLR protein levels via the activation of phosphoinositide 3-kinase (PI3K)/protein kinase B (Akt) pathway [29]. The involvement of P13K/Akt pathway was demonstrated by the disappearance in hypocholesterolemic effects in the presence of wortmannin, a potent inhibitor of P13K [35]. 

LDPs are also able to suppress lipid synthesis and accumulation and improve antioxidant and anti-inflammatory status after oral ingestion of LPHs by animal model of obesity [127]. Another useful observation has been provided by a recent clinical study that has shown that the consumption of 30 g/day lupin protein for three months reduced the plasma PCSK9 level by 12.7% [125]. The molecular mechanism of this modulation has been investigated using HepG2 cells, demonstrating that either the peptic or the tryptic lupin hydrolysates decrease the mature PCSK9 protein levels and/or its secretion into the extracellular environment. A subsequent investigation has indicated that LILPKHSDAD is responsible for this mechanism of action, since it reduces the mature PCSK9 protein level and its secretion through its ability to decrease its transcription factor, HNF-1α [125]. In addition, LILPKHSDAD is also able to inhibit the protein-protein interaction (PPI) between PCSK9 and LDLR with an IC_50_ equal to 1.6 µM. A bioinformatic tool, using in silico docking model has enhanced the understanding of the interaction of LILPKHSDAD with the LDLR binding site of PCSK9, thus showing how the peptide impairs the PPI between these two important proteins [123]. Interestingly, LILPKHSDAD is the first hypocholesterolemic peptide characterized by this dual inhibitory activity. 

GQEQSHQDEGVIVR is another bioavailable peptide from lupin β-conglutin, which is able to exert a direct inhibition of the PCSK9/LDLR PPI, even though with a lower potency [123,126]. However, only GQEQSHQDEGVIVR inhibits the PPI between the LDLR and the dangerous gain-of-function of PCSK9 mutant named, PCSK9^D374Y^ [128], which is responsible of a severe form of hypercholesterolemia. Furthermore, this peptide is also able to reduce the mature PCSK9^D374Y^ protein level in HepG2 cells, showing unique features among food-derived peptides endowed with cholesterol-lowering activity [129].

Based on these observations and considering that PCSK9 inhibitors have been added in the most recent guidelines for managing hypercholesterolemic conditions [130], the ability of lupin peptides to inhibit HMGCoAR activity and PCSK9 signaling and upregulate LDLR protein levels imply that LDPs are attractive candidate for nutraceutical developments for promoting cardiovascular disease prevention. 

One target for controlling hyperglycemic conditions, which is recently gaining scientific attention, is the inhibition of intestinal dipeptidyl peptidase-IV (DPP-IV) activity, leading to the enhancement of glucose-mediated stimulation of insulin secretion by the β-cells of the pancreas [131]. Based on its traditional use in treating diabetes, lupin seed-fortified beverages have been demonstrated to improve glycemic control in diabetes [132]. Notably, LTFPGSAED was reported to moderately inhibit DPP-IV activity in Caco-2 cells (with IC_50_ value of 207.5 µM), and the inhibitory activity in human serum and in human intestinal Caco.2 cells was sustained over 4 h [30], suggesting that the peptide is resistant to intestinal protease and serum peptidases. 

### 2.6. Anti-Hypertensive Effect

The renin-angiotensin-aldosterone system (RAAS) plays a main role in the regulation of blood pressure, fluid volume, sodium level among others, and its overactivity or disruption of any of the component has been identified in many cases of hypertension and the consequent end-organ damages [133,134,135]. This has made RAAS a target for the many drugs used in controlling blood pressure and preventing its complications. This class of drugs act by inhibiting the release of renin, the conversion of angiotensin I to angiotensin II via inhibition of angiotensin-converting enzyme (ACE) and/or blocking of angiotensin receptors (AR), especially angiotensin receptor-1 (AR-1) and mineralocorticoid receptor (MR) that interact with angiotensin II to promote aldosterone secretion [136]. Several natural products are currently being explored as potential anti-hypertensive agents, including plant-polyphenols and protein-derived peptides [137,138,139]. Hydrolysates of lupin proteins exhibited potential anti-hypertensive effects in an in vitro set-up by inhibiting ACE activity with IC_50_ value of 226 µg/mL [4]. It has also been shown that enzymatic products of of proteins isolated from seeds of different lupin species, including *Lupinus luteus, L. albus,* and *L. angustifolius* have ACE-inhibitory activities and that the use of combined hydrolytic enzymatic systems improves the anti-hypertensive properties of the peptides [5]. This implies that the use of combined proteases might increase the production of low molecular weight peptides (LMWPs). LMWPs have been reported to possess better hypoglycemic effect compared to high molecular weight peptides (HMWPs), partly due to their higher potential of permeating the intestinal epithelium and to reach their targets relative to HMWPs [140,141]. 

In a recent investigation, Australian researchers observed that LMW fractions of LPHs (2–5 kDa) inhibited ACE activity (IC_50_ values of 450 to 600 μg/mL) [33]. In addition, LTFPGSAED with hypoglycemic and hypolipidemic properties were tested for ACE-inhibitory activities in both intestinal Caco-2 and renal HK-2 cells. The nonapeptide inhibited ACE activities in the two cells with IC_50_ values of 13.7 μM and 79.6 μM, respectively. The observation suggests that intestinally-generated metabolites may have contributed to the higher ACE-inhibitory activity of the peptide in intestinal cells than renal cells. Further investigation showed that intestinal degradation of LTFPGSAED by peptidases (such as DPP-IV) generated shorter peptides such as TFPGSAED and LTFPG, confirming that these metabolites contribute to the ACE-inhibitory activities of the parent peptide. This observation complements a previous report by Aluko et al. [142] that LTFPG isolated from pea seed provicilin (30 mg/kg) possess hypotensive effects in spontaneously hypertensive rats (SHRs) partly by inhibition of ACE and renin activities as demonstrated in an in vitro study. The biological activities observed after oral ingestion of peptides may not be a direct effect of the intact peptide ingested but partly by the metabolite(s) of the parent peptides. For instance, upon total degradation of the multifunctional nonapeptide, LTFPGSAED to LTFPG, there was a loss of DPP-IV inhibitory activity while ACE- and HMGCoAR-inhibitory activities were improved and retained, respectively [32]. 

**Figure 1:**
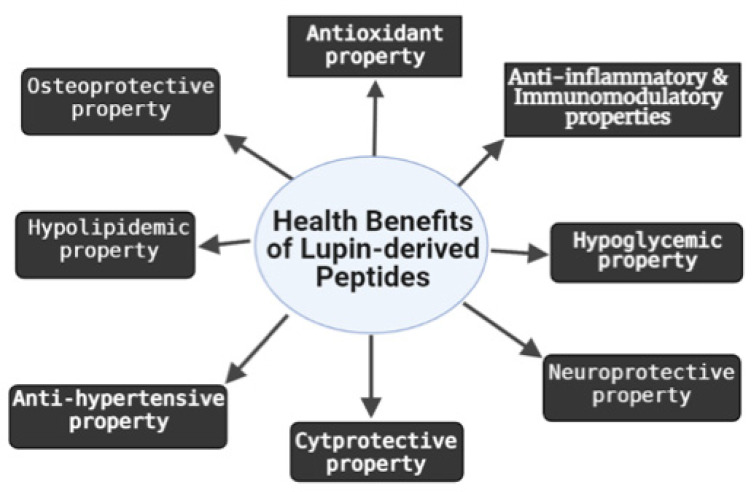
Summary of biological activities of lupin-derived peptides.

The biological activities of lupin hydrolysates and peptides isolated from them are summarized in Table 1 and Figure 1.

## 3. Structure-Activity Relationship of Bioactive LDPs

The preceding paragraphs have shown that interesting LPDs have been shown to exert many biological effects. For attaining fruitful applications, it would be important to identify which function as inhibitors of known target enzymes and which are involved in cellular pathways related with specific diseases. Basically, behind the hypocholesterolemic activity of peptides, different mechanisms of action may occur. In particular, in order to function as a competitive inhibitor of HMGCoAR, a peptide should mimic the hydroxymethylglutaryl moiety. To achieve this goal, the conformation and the side chain groups play a more important role than the total hydrophobicity. Moreover, the correlation of the inhibitory activity with the peptide length is still unclear. The lupin peptides LTFPGSAED, LILPKHSDAD, and YDFYPSSTKDQQS contain at least one Pro residue, which mimics the nicotinamide moiety of NADPH, which is the enzyme co-factor [143,144]. Moreover, it has been established that a Leu, Ile and/or Tyr residue at the N-terminus and a Glu residue at the C-terminus play important roles for the peptide inhibitory property [143,144]. Indeed, LTFPGSAED, LILPKHSDAD peptides and their metabolites (TFPGSAED, LTFPG, ILPKHSDAD) and satisfy these features. However, only peptide LTFPGSAED comprises two negative charged side chains at C-terminal tail that improve its ability to interact with the receptor site and make it the best HMGCoAR inhibitor. Recently, it has been figured out that during an absorption study of LTFPGSDAD, Caco-2 cells produce a metabolite, LTFPG, which it is less active than the parent peptide as a HMGCoAR inhibitor [145]. This result clearly confirms the importance of the negative charged side chains at C-terminal tail for achieving an effective enzyme inhibition. LTFPGSAED displays the ability to drop the DPP-IV activity, a recent in silico study confirm the ability of this peptide to interact with the enzyme active side predicting the binding mode [146]. In addition, some years ago, a patent had reported the structures of 21 peptides capable of inhibiting DPP-IV activity [147]. They have a hydrophobic character, a length varying from two to eight amino acid residues, and contain a Pro residue within their sequences, which is located at the first, second, third, or fourth N-terminal position. Besides, the Pro residue is flanked by Leu, Val, Phe, Ala, and Gly. In light with these observations, the fourth N-terminal residue of LTFPGSAED comprises a Pro, which is flanked by a Phe residue. Moreover, this lupin peptide is mostly composed of hydrophobic amino acid residues (Ala, Gly, Ile, Leu, and Pro) [146].

Many physical-chemical factors may influence the ability of peptides to exert antioxidant activity. In facts, although certain aspects of the structure-function relationship of antioxidant peptides are still poorly understood [148], it has been suggested that chain length, amino acid type, amino acid composition, and amino acid sequence, the location of specific amino acids in a peptide chain may be critical issues for exerting the antioxidant property [149]. In this context, short peptides may be often potent antioxidants. Literature indicates that, besides containing hydrophobic amino acids, such as Leu or Val, in their N-terminal regions, peptides containing nucleophilic sulfur-containing amino acid residues (Cys and Met), aromatic amino acid residues (Phe, Trp, and Tyr) and/or the imidazole ring-containing His are generally found to possess strong antioxidant properties [150]. In light with these considerations, the lupin FVPY completely satisfy the above-mentioned features, in facts this short and hydrophobic peptide comprises two aromatic amino acid residues (Phe and Tyr) and also a Val-residue close to its N-terminal region. 

## 4. Transepithelial Transport, Biostability and Bioavailability of LDPs

To elicit their biological responses, peptides must be resistant to proteolytic hydrolysis by intestinal proteases and peptidases and be transported to their target organs in their functional form [151,152]. The various mechanisms through which peptides are transported across the intestinal compartment into circulation are highlighted below. The first is paracellular diffusion, which involves the movement of molecules through water-filled pore between cells. This mechanism is regulated by tight junction and is dependent on physicochemical properties of a peptide. The second mechanism is transcellular passive diffusion. This involves the transport of peptides in a concentration-based and energy-independent manner. This mechanism is dependent on the size, charge and hydrophobicity of the peptide. The third is the transcytosis, an energy-dependent transport of material from one side of polarized cell to the other. This mechanism favors the transport of long-chain and hydrophobic peptides. The fourth mechanism is carrier-mediated transport which involves the movement of peptides against their concentration gradient. This process is facilitated by specific cell membrane proteins and favors short-chain peptides [153,154]. While some bioactive peptides are linearly transported in an intact form across the intestinal epithelium to bind to their target and elicit biological response, others act as “prodrugs” and as such are transformed to their active metabolites during transport across the intestine by proteases and in circulation by serum peptidases [85].

LDPs have been shown to have sufficient cellular bioavailability as demonstrated in differentiated Caco-2 cells [155]. Notably, a multidisciplinary study has been carried out in order to characterize the ability of LPHs obtained using pepsin and trypsin, to be transported across differentiated human intestinal Caco-2 cells. Results indicate that, eleven and nine peptides, respectively, from tryptic and peptic hydrolysates are found to linearly permeate the intestinal epithelium [155]. In recent studies, it has been clearly demonstrated that the transepithelial transport of peptides is highly influenced by the presence of other peptides [156] For example, using mature Caco-2 cells, a recent study has evaluated the transepithelial transport rate and mechanism of LDPs, LILPKHSDAD and its metabolite, LPKHSDAD when examined alone and/or mixed together other lupin peptides, YDFYPSSTKDQQS and LTFPGSDAD [29]. The presence of other peptides favors the transport of LDPs possibly by increasing their stability and impairing their enzymatic degradation. Since peptide size, hydrophobicity and charge are major factors affecting the route through which peptides are transported and absorbed [156], the transepithelial transport of LILPKHSDAD is likely via passive transcellular diffusion and/or transcytosis, since it is a decapeptide with a net charge of -1 and hydrophobicity of +17 kcal/mol. The ability of wortmannin, a potent transcytosis inhibitor to impair the transport mechanism of LILPKHSDAD across caco-2 cell layer strongly suggests that transcytosis is its most favorable transport mechanism. On the other hand, the inability of wortmannin to impair the transport of LPKHSDAD across Caco-2 cells showed that its transport is independent of transcytosis [29]. In a similar experiment, the rate of absorption of LTFPGSAED and its metabolite, LTFPG, in culture Caco-2 cells were improved when mixed with YDFYPSSTKDQQS and LILPKHSDAD [32]. Considering the opinion of Sun et al. [157] and Udenigwe et al. [158] that other components of the matrix via which some peptides are delivered influence their biostability, bioaccessibility and bioavailability, all of which impact negatively on their bioactivities, the effect of food matrix on the bioactivities of LDPs should be investigated. This is to select the best matrix for delivering LDPs to achieve the desired health benefits.

## 5. Safety Concerns of LDPs

Considering that lupin protein-based products such as peanuts and other snacks are being associated with allergic reactions in some individuals, specifically the immunoglobulin (Ig) E-type hypersensitivity reaction [159], it is possible that some individuals may also be allergic to LDPs. The α/β-conglutins proteins in lupin have been implicated in the vast majority of allergic reaction associated with lupin, with minor contribution by γ/δ-conglutins proteins [160]. Storage and processing using trypsin enzyme were shown to reduce the allergenicity of lupin proteins [161], suggesting that alteration in the native structure of the protein during storage and processing impacts on the amino acid sequence responsible for immunogenicity. Further study should identify the specific amino acid residues/sequence that binds to IgE (antigenic determinants) to elicit immune response. The ability of LPHs to demonstrate anti-inflammatory and immunomodulatory properties [93] in human peripheral blood cells, and because beverage prepared using lupin proteins elicited no immune reaction on participants of a recent human clinical trial [42] implies that the antigenic determinant(s) of lupin protein may have been destroyed during enzymolysis. Based on the above, further research is needed to clarify the best hydrolytic method for preparing LDPs that will eliminate the allergenicity of the intact proteins without tampering with their bioactivity. It is also recommended that α/β-conglutins proteins, the major culprits in lupin allergy, should be separated from the crude protein isolate during processing to abolish their ability to induce allergic reactions.

## 6. Conclusions and Future Prospects

Lupin protein hydrolysates and peptides derived from them have been shown to exhibit antioxidant, anti-inflammatory, immunomodulatory, hypolipidemic, hypoglycemic, anti-hypertensive, osteoprotective, neuroprotective, and cytoprotective properties. These multifunctional health benefits make LDPs excellent candidates for the development of functional foods. Some of the limitations in the bioactivities of LDPs, as observed in many other peptides, include limited biostability, bioavailability and bioaccessibility. A number of strategies such as incorporation of peptides into biocompatible vehicles, adoption of preparation methods with limited negative impact on the natural properties of the peptides and incorporation of peptide-based nutraceuticals with inhibitors of proteases and peptidases to enhance their stability during transepithelial transport and bioavailability are recommended for future investigation. Considering that majority of the bioactivities reported of LDPs are based on in vitro and cell culture studies, more efforts need to confirm these biological activities in animal models and human clinical studies for the development of functional foods or dietary supplements. In addition, increasing investigations will be also useful to establish a good correlation between the structure and the function as well as the assessment of absorption, distribution, metabolism, excretion, and toxicity (ADMET) of the peptides and their derivatives. 

## Figures and Tables

**Table 1:** Summary of biological activities of lupin-derived peptides.

Lupin Hydrolysates and Peptides	Biological Activities	References
Non-purified low molecular weight peptides	ACE-inhibitory and hypoglycemic effects	[4,5,33]
LTFPGSAED	Hypoglycemic and insulin-mimetic properties	[15,28]
GPETAFLR	Anti-osteoclastogenic effect in LPS-induced human monocyte-derived osteoclasts. The peptide also improved GSH synthesis and suppress intracellular ROS generation	[28]
IQDKEGIPPDQQR	Suppressed the expression and protein levels of pro-inflammatory cytokines and their receptors	[31]
LTFPGSAED, TFPGSAED and LTFPG	DPPIV, HMGCoAR and ACE-inhibitory activities	[32]
Pancreatin hydrolysates	ACE inhibitory and antibacterial (*Bacillus cereus* and *Staphylococcus aureus*) activities	[33]
FVPY	Antioxidant and lipid peroxidation properties	[75]
Unspecified low molecular weight peptides	Radical scavenging and ferric reducing antioxidant properties via Keap-1/Nrf2 signaling pathways	[80]
Hydrolysates	Anti-inflammatory and immunomodulatory effects	[93]
Izyme AL and Alcalase 2.4 L-generated hydrolysates	Increased the production of anti-inflammatory cytokines while inhibiting the production of NO and pro-inflammatory cytokines in LPS-induced mouse RAW 264.7 macrophages	[94]
GPETAFLR	Suppressed the expression and protein levels of pro-inflammatory cytokines and their receptors in LPS-induced retinal pigment epithelium cells	[94]
GPETAFLR	Promoted the differentiation of M2 phenotype of peripheral macrophage system that enhances anti-inflammatory processes while inhibiting M1 phenotype of peripheral macrophage system that enhance pro-inflammatory processes	[114,115]
LILPKHSDAD	Suppressed cholesterol synthesis by inhibiting HMGCoAR via AMPK pathway, and increased LDLR level via upregulation of SREBP-2 expression	[125]
